# Off the beaten track: the molecular structure of long-term memory: three novel hypotheses—electrical, chemical and anatomical (allosteric)

**DOI:** 10.3389/fnint.2015.00004

**Published:** 2015-01-29

**Authors:** John Smythies

**Affiliations:** ^1^Department of Psychiatry, Center for Brain and Cognition, University of California San DiegoLa Jolla, CA, USA; ^2^Department of Psychiatry, University of Alabama at BirminghamBirmingham, AL, USA

**Keywords:** cytoskeleton, microtubules, tubulin, Liquid State Machine, tau, heteroreceptor complexes, allosteric interactions, bar codes

This opinion paper reviews three hypotheses relating to the possible molecular events involved in the formation of long-term memories that go beyond the classical theory, which is confined to strengthening Hebbian synapses. The aim of this paper is not to write detailed critiques of these three hypotheses but to present, for the first time in one location, their essential components to engage the interest of the reader in these new ventures.

## Hypothesis 1. electrical activity in the cytoskeleton

The microtubule-actin filament matrix inside neurons may comprise an integrative intraneuronal network by binding to and linking ion channels and neurotransmitter receptors, scaffolding proteins and their adaptors, motor proteins, microtubule-associated proteins (MAPs) including MAP2 and tau, and other linking proteins (Plankar et al., 2012). There is a considerable body of evidence, reviewed by Priel et al. (2010), that long-term memory formation may involve changes in the cytoskeleton.

One electrical hypothesis to explain this suggests that the matrix acts as a species of high-dimensional non-linear dynamic assembly called a Liquid State Machine (LSM) (Priel et al., 2010). According to this hypothesis this matrix receives electric signals from synapses and ion channels, partly via calcium signaling, targeted phosphorylations and MAPs, and integrates these according to its own non-linear dynamics. Its output directly regulates ion channels and receptors, where the main degrees of freedom are related to the amount of electric flow along each MT. The current dynamic state of the system continues to evolve as new input signals arrive. The hypothesis suggests that the intraneuronal matrix affects the neuronal response by electrically modulating the activity of voltage-depended ion channels, or by inducing cytoskeletal reorganization via signal transduction pathways (Plankar et al., 2012). This electric signaling flow may be carried along the MT by fluctuations in counter-ion ionic clouds. MTs contain an excess of negatively charged aminoacid residues that will attract positively charged ions that self-organize as a dynamic counter-ion cloud around the MT (Priel et al., 2010). Plankar et al. (2012) stress the importance of the modulation of synchronized potentials by this matrix. They conclude

“At the network scale, the long-range interactions within these matrices could help explain—e.g., via resonant coupling with the membrane potential fluctuations and network oscillatory entrainment—the experimentally observed rapid transitions of the phase and the carrier frequency, defining distinct patterns of synchronized neuronal oscillations, which have as yet not been conclusively explained by the classical models of functional connectivity.”

## Hypothesis 2. chemical changes in the cytoskeleton

I suggest here a chemical hypothesis for the possible role of microtubules in long-term memory formation. Neurotransmitter-based signaling to the microtubule cytoskeleton regulates downstream microtubule function through several mechanisms. These include tubulin posttranslational modification, binding of microtubule-associated proteins, release of microtubule-interacting second messenger molecules, and regulation of tubulin expression levels (Gardiner et al., 2011). Microtubules are highly dynamic structures and are known to rapidly invade dendritic spines in mature central nervous system neurons (Hu et al., 2008). These authors report that they found that only a small percentage of spines are targeted at a time, and that all targeting events are transient, averaging only a few minutes. Nevertheless, over time, many spines on a dendrite are targeted by microtubules. Importantly, the authors report that increasing neuronal activity enhances both the number of spines invaded by microtubules and the duration of these invasions.

In this hypothesis the cytoskeleton can be regarded as having two components. The first (A) consists of actin-microtubule networks connected to primary information transmitting receptors including glutamate and GABA. The second (B) consists of actin-microtubule networks connected to receptors for many neuromodulators including dopamine. Gardiner et al. (2011) provide an exhaustive list of these. The two networks are densely cross-linked by MAPs.

All we have to suppose is that the differentiation and growth of network A, involved in the formation of long-term memories, is promoted by simultaneous, reinforcement-linked, intermittent activity in the growth and dynamic state of polymerization of network B. This restless growth, so vividly described in spines by Hu et al. (2008), may be transmitted chemically to A by specific MAPs, and possibly by calcium waves. This may lead in turn to the rapid growth and differentiation of selected MT segments. In this way short-term memories carried by networks of transient potentials at the synapse level could be transformed by reinforcing signals from B into long-term memories carried by structural growth of the cytoskeleton.

In support of such an hypothesis, Lebel et al. (2009) found that dopamine D1 receptor activation induces phosphorylation of the microtubule protein tau (that is involved in microtubule stabilization) via cyclin-dependent kinase 5 (cdk5) and glycogen synthase kinase 3β (GSK3) signaling pathways (Lebel et al., 2009; Lebel and Cyr, 2011). As phosphorylation of tau is associated in membrane destabilization perhaps the effect of dopamine activation needs to start with destabilization of the cytoskeleton of the existing network before the new framework can be imprinted, as suggested for their own electrical hypothesis by Priel et al. (2010). Alternatively other dopamine receptors may be involved about which there is at present no data.

## Hypothesis 3. allosteric anatomy

An elegant molecular-structural hypothesis of long-term memory formation has been suggested by Fuxe et al. (2014). This hypothesis proposes that intramembrane allosteric interactions between different receptor molecules (receptor protomers) in postsynaptic homo- and hetero-receptor complexes including GPCRs, play a key role in modulating the manifold functions especially of the heteroreceptor complexes of the post-synaptic density and its perisynaptic regions that lead to the laying down of LT memories. The mechanism proposed also includes secondary changes in the prejunctional receptor complexes (probably involving information carried across the synapse by agents like exosomes), and transformation of parts of the heteroreceptor complexes into unique transcription factors that can generate new proteins. These can consolidate in particular the heteroreceptor complexes into long-lived complexes that carry long-term memories.

This entails the following process. When a transmitter or neuromodulator binds to its receptor it evokes a conformational (allosteric) change in the receptor protomer. Since this protomer is part of a heteroreceptor complex the allosteric information will pass in the receptor interface to the other receptor protomers of the complex and change their recognition, pharmacology, signaling and trafficking including GCPRs, ion channel receptors, receptor tyrosine kinases. Thus, the allosteric waves will, via the receptor interface, induce conformational changes in the other receptor protomers of the heteroreceptor complex and change their function. This process then triggers two other consequences.

1. A signal is carried back across the synapse (via soluble molecules or carried by exosomes) that induces changes in the presynaptic heteroreceptor complex to help maintain the spatio-temporal pattern of multiple transmitter release from presynaptic vesicles to be learned by the post-perisynaptic membrane. This pattern is learned through a reorganization of the various homo- and hetero-receptor complexes in the post-perisynaptic membrane into formation of novel “bar-codesll” represented anatomically by the novel heteroreceptor complexes formed, and physiologically by their new balance with the corresponding homoreceptor complexes. In this way a molecular engram for short-term memory is created.
2. Long-term memory develops when unique transcription factors are produced from parts of the heteroreceptor complexes that enter the nucleus and generate new proteins that can bind to and consolidate the new heteroreceptor complexes and stabilize the “bar-codes” into long-term memories.

The cumulative effect produced by this mechanism is the laying down of stable molecular complexes in the synapse and presynaptic regions that carry the information pertaining to long-term memories.

To summarize: the repeated temporal pattern of a transmitter code in the synaptic cleft can produce a unique firing pattern in the postsynaptic nerve cell that leads to the formation of a long-term memory. This takes place through the reorganization and thus resetting of multiple heteroreceptor complexes in the post and perisynaptic membrane that become consolidated by the formation of unique transcription factors in the postsynaptic neuron. The mechanism proposed is that coded sensory and cognitive information is delivered to the neuron via rapidly fluctuating complex spatio-temporal patterns of release of a variety of synaptic vesicles. This is translated by modulations of the microanatomical structure of postsynaptic multireceptor complexes into semipermanent information stored as long-term memories, and manifested in behavior. This is accompanied by feedback that modulates the function of presynaptic heteroreceptor complexes to maintain the spatio-temporal transmitter release pattern to be learned. The latter may then precisely set the recruitment of vesicles to the plasma membrane and the amount of extracellular release of neurotransmitters and modulators from vesicles located in the terminal varicosity. This process is linked to the pattern of action potentials that reach and depolarize the terminal membrane.

The cumulative effect produced by this complex molecular mechanism is the laying down of stable and multiple postsynaptic heteroreceptor complexes in the synapse that carry the information pertaining to long-term memories.

## Strengths and weaknesses of the three hypotheses

One potential strength of the first two hypotheses is that both are based on a quantity of data from many different laboratories. Another is that they deal with phenomena that are familiar to all neuroscientists. These phenomena include signaling involving voltage-dependent ion channels, chemical and electrical transduction pathways, electrically charged residues in proteins, synchronized potentials, non-linear dynamics and others. One potential weakness in both is that the cytoskeleton already has one well-defined function i.e., acting as rails for the transport of large molecules and organelles by protein engines such as kinesin and dynein, and it is not clear how this mechanical transport system would interact with the electrical and chemical signaling systems along these same tracks proposed by the these two hypotheses. This issue needs further experimentation to resolve.

The concept of signaling by intramembrane allosteric interactions between protein molecules proposed by the third hypothesis is very novel and possibly unfamiliar to most neuroscientists. The concept that information can be stored in neurons by “bar codes” of electrical excitations is even more novel. However, novelty is no bar to excellence and quite new lines of research may develop from this source. For example, the use of NeuronAnalyzer2D technology, that allows the ability to extract the subcellular distribution of distinct biomolecules along neurites, may throw some light on this question (Misiak et al., 2014).

## Conclusion

This opinion paper presents the essential features of three novel hypotheses—one electrical, one chemical and one anatomical (allosteric)—concerning the molecular mechanism involved in laying down LT memories. These hypotheses are not necessarily mutually exclusive. An example of such an interaction between chemical and microanatomical factors is given by Craddock et al. (2012). These authors base a theory of LT memory formation on the interaction of molecules of Ca^2+^-calmodulin dependent kinase II (CaMKII) with molecules of tubulin. CaMKII is a dodacameric holoenzyme containing 2 hexagonal sets of 6 kinase domains. The authors state—

“Each kinase domain can either phosphorylate substrate proteins, or not (i.e., encoding one bit). Thus, each set of extended CaMKII kinases can potentially encode synaptic Ca^2+^ information via phosphorylation as ordered arrays of binary “bits.” Candidate sites for CaMKII phosphorylation-encoded molecular memory include microtubules (MTs), cylindrical organelles whose surfaces represent a regular lattice with a pattern of hexagonal polymers of the protein tubulin.”

Only further research can determine which, if any, of these hypotheses (in whole, in part or in combination) is correct.

### Conflict of interest statement

The author declares that the research was conducted in the absence of any commercial or financial relationships that could be construed as a potential conflict of interest.
